# Laparoscopic low anterior resection for rectal cancer associated with Leriche syndrome: a case report

**DOI:** 10.1186/s40792-022-01438-1

**Published:** 2022-04-27

**Authors:** Ryo Nakanishi, Atsuko Tsutsui, Hiroto Tanaka, Kohei Mishima, Chie Hagiwara, Takahiro Ozaki, Kazuharu Igarashi, Satoru Ishii, Nobuhiko Okamoto, Kenji Omura, Go Wakabayashi

**Affiliations:** Department of Surgery, Ageo Central General Hospital, 1-10-10 Kashiwaza, Ageo, Saitama 362-8588 Japan

**Keywords:** Rectal cancer, Computed tomography angiography, Leriche syndrome

## Abstract

A 78-year-old male presented with a positive fecal occult blood test. Rectal cancer was detected during lower gastrointestinal endoscopy, and further investigations led to a diagnosis of cT1N0M0 cStage I (UICC classification, 8th edition). Preoperative contrast-enhanced computed tomography (CT) showed that the patient also had Leriche syndrome, which is associated with reduced blood flow to the rectum that may result in ischemic anastomosis during rectal cancer surgery with anastomotic reconstruction. The inferior epigastric arteries often function as collateral pathways to the lower limbs in patients with Leriche syndrome; therefore, care is needed to avoid vascular damage during trocar insertion when performing laparoscopic surgeries. We herein described a case of safe laparoscopic low anterior resection in a rectal cancer patient with Leriche syndrome using vascular architecture images obtained by preoperative CT angiography.

## Background

Leriche syndrome, also commonly referred to as aortoiliac occlusive disease, causes impotence and claudication [1, 2]. Hypertension, diabetes mellitus, and a history of smoking are risk factors for Leriche syndrome. The following issues need to be considered when performing rectal cancer surgery on patients with Leriche syndrome. Occlusion of the internal iliac artery or inferior mesenteric artery (IMA) reduces blood flow to the rectum and may be associated with ischemic anastomosis during rectal cancer surgery. Furthermore, since blood flow to the lower limbs is supplied by collateral arteries, such as the inferior epigastric artery and deep circumflex iliac artery [3], care is needed to avoid vascular damage during port insertion when performing laparoscopic surgeries on patients with Leriche syndrome. In the present case, laparoscopic surgery was conducted for rectal cancer complicated by Leriche syndrome based on images of the vasculature obtained by preoperative computed tomography (CT) angiography and intraoperative fluorescence imaging with indocyanine green (ICG).

## Case presentation

**Fig. 1:**
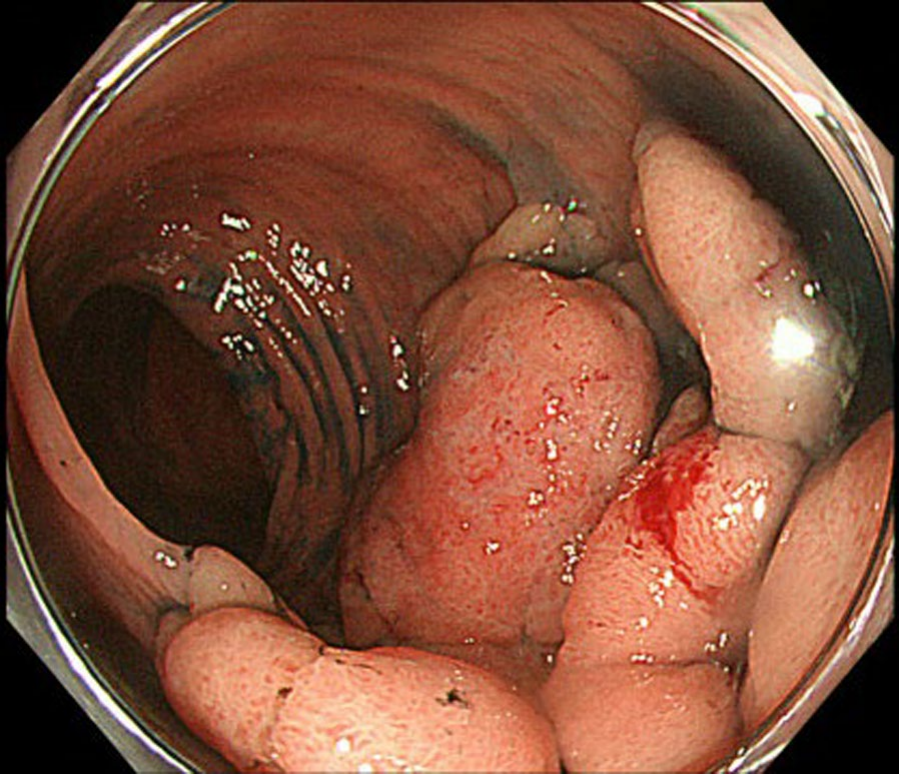
Rectal cancer (0–IIa + IIc) was detected 10 cm from the anal verge and the lesion site was inked (white arrow). Biopsy revealed well-differentiated adenocarcinoma

**Fig. 2:**
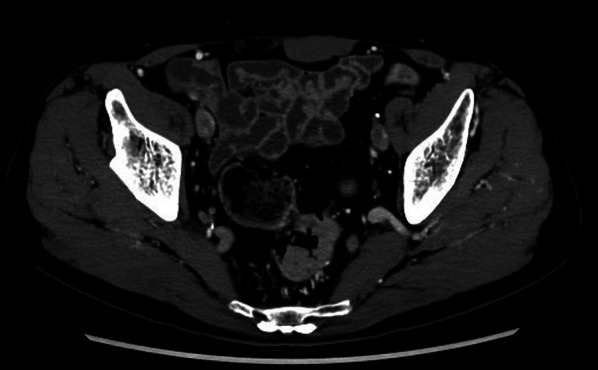
Abdominal contrast-enhanced computed tomography (CT) revealed a thickened rectal wall, but no obvious distant metastasis (white arrow)

**Fig. 3:**
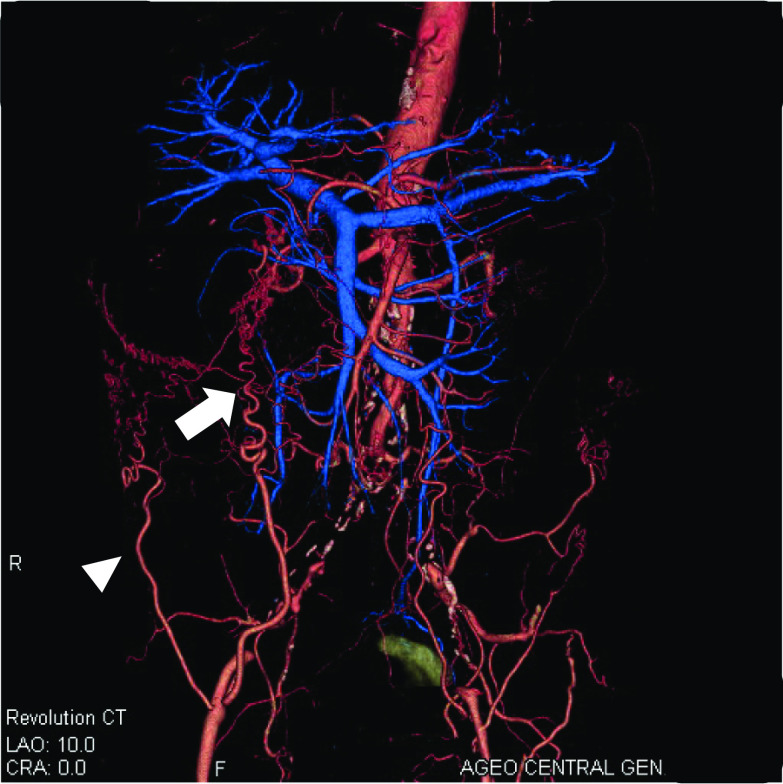
The abdominal aorta was occluded from below the renal artery bifurcation to around the common iliac artery bifurcation. Blood flow to the lower limbs was supplied through the inferior epigastric arteries and deep circumflex iliac artery (white arrow)

**Fig. 4:**
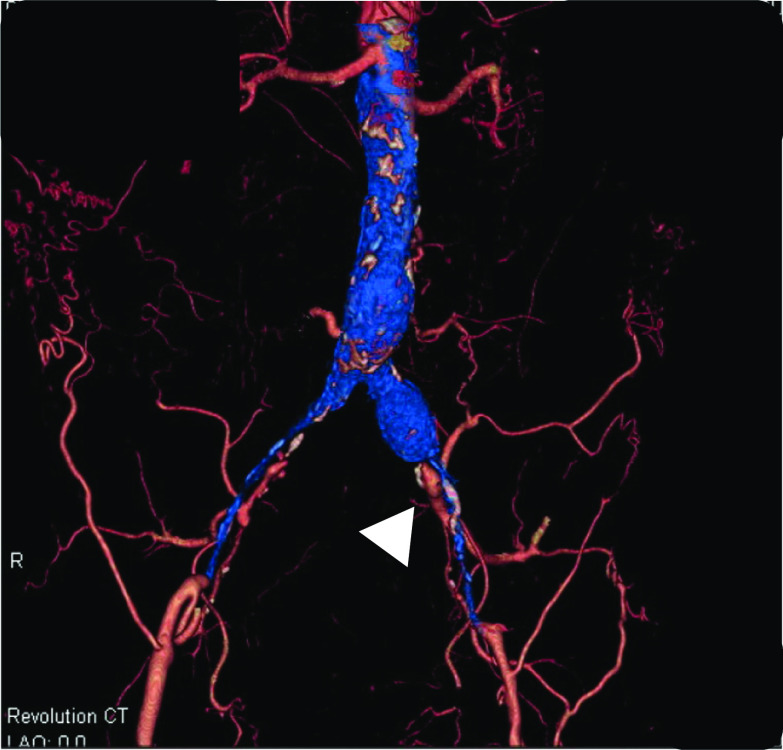
CT angiography revealed the left internal iliac artery was also not completely occluded (white arrowhead)

**Fig. 5:**
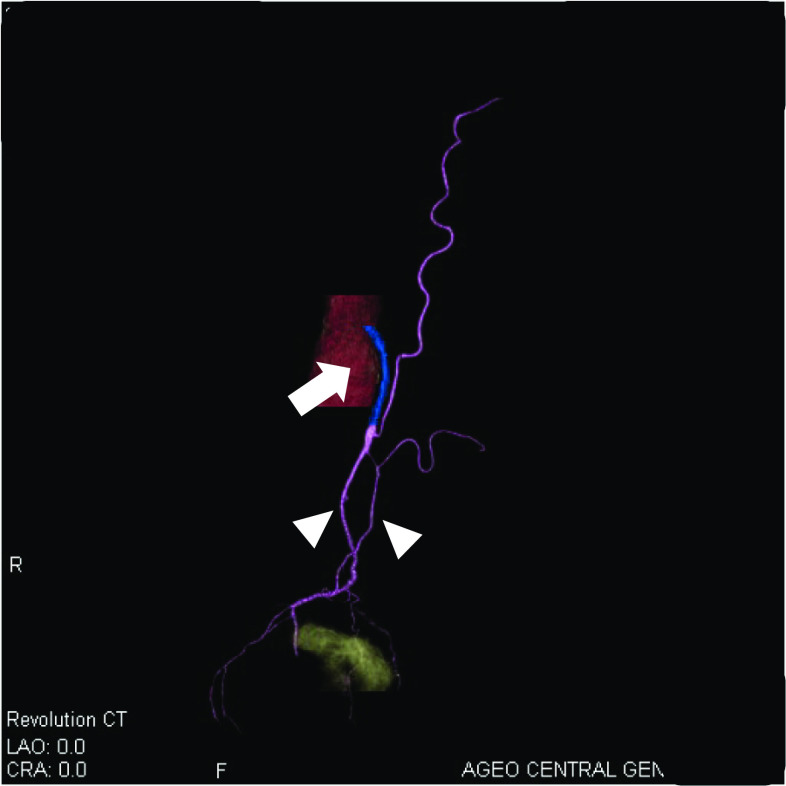
CT angiography revealed occlusion of the aorta just below the origin of the inferior mesenteric artery (white arrow) and the dominant vessels of the tumor were identified as SRA and S2 (white arrowhead)

**Fig. 6:**
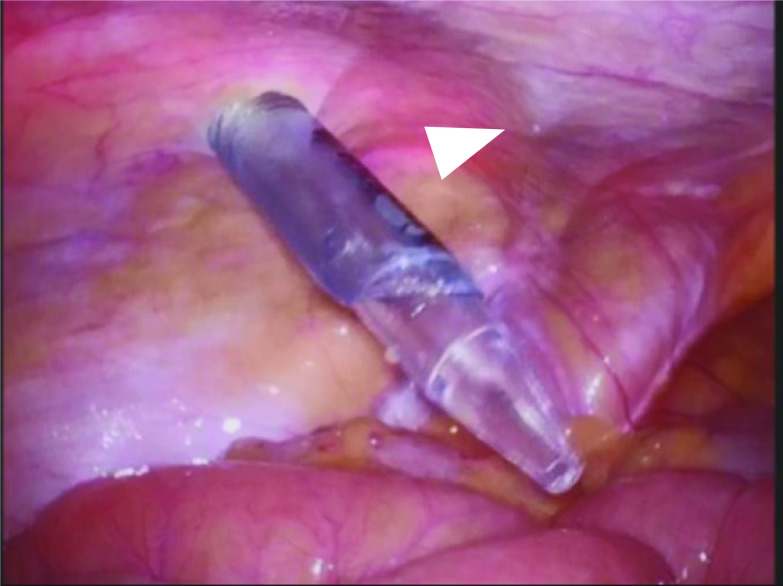
Intraoperative port insertion finding. The port is inserted avoiding the inferior epigastric artery. The white arrowhead shows an epigastric artery

**Fig. 7:**
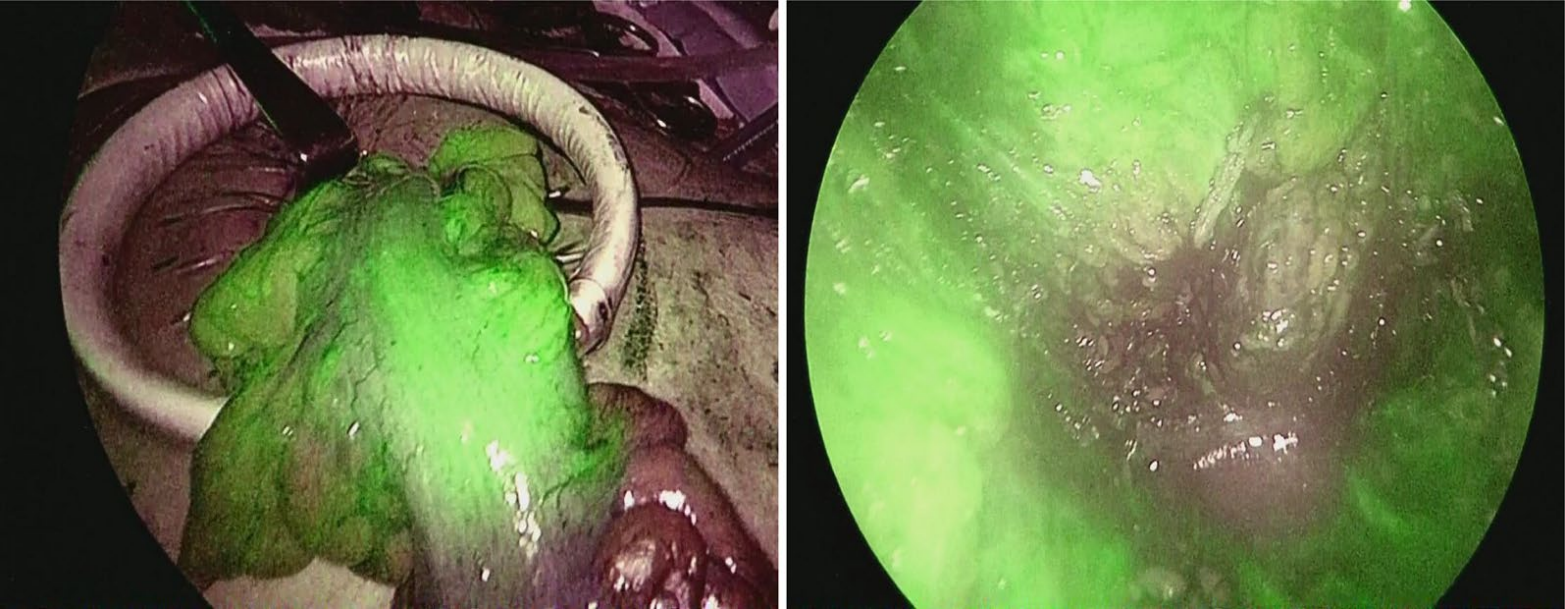
During the creation of the anastomosis, indocyanine green-based fluorescence imaging was used to confirm that intestinal blood flow had been preserved (we administered 12.5 mg of ICG and confirmed that the anastomosis was contrasted in 27 s). The left side is the indocyanine green-based fluorescence imaging of the oral side of the anastomosis, while the right side shows that of the anal side of the anastomosis

The patient was a 78-year-old male with a history of hypertension and previous appendectomy. He also smoked 30 cigarettes a day for 30 years. His family history was unremarkable. He visited our department after a positive fecal occult blood test and was referred for surgery because colonoscopy revealed rectal cancer. Blood chemistry and cardiac and pulmonary function tests showed normal values. Colonoscopy revealed 0–IIa + IIc rectal cancer 10 cm from the anal verge, and the lesion site was inked (Fig. 1). Abdominal contrast-enhanced CT (Fig. 2) showed a thickened rectal wall, but no obvious distant metastasis. The abdominal aorta was occluded from below the renal artery bifurcation to around the common iliac artery bifurcation, causing so-called Leriche syndrome. A left common iliac artery aneurysm was also detected. Blood flow to the lower extremities was supplied by the inferior epigastric artery and deep circumflex iliac artery as collateral blood vessels (Fig. 3). Blood flow to the rectum was supplied from the superior mesenteric artery to the IMA via the middle colonic artery and Riolan’s arcade due to occlusion of the IMA. The left internal iliac artery was also not completely occluded. Therefore, the left internal iliac artery supplied blood flow to the rectum (Fig. 4). The major feeding vessels of the tumor were identified as the superior rectal artery (SRA) and second sigmoid artery (S2) (Fig. 5). According to the Union for International Cancer Control (UICC) classification, 8th edition, the tumor was categorized as clinical stage I (cT1N0M0). The patient underwent laparoscopic low anterior resection and D2 lymph node dissection. Under general anesthesia, he was placed in the lithotomy position. A 10-mm camera port was created at the umbilicus, and pneumoperitoneum pressure was set to 8 mmHg. Laparoscopy did not reveal any liver metastasis, peritoneal dissemination, or adhesions. The tumor was identified at the inked site in the rectum. A further four ports were inserted, with care being taken to avoid damaging the distended inferior epigastric artery, and normal port placement was achieved (Fig. 6). To reduce the postoperative complications, the left colic artery (LCA) was preserved and the major tumor-feeding vessels; i.e., the SRA and S2, were dissected. ICG-based fluorescence imaging was used during the creation of the anastomosis to confirm sufficient intestinal blood flow on the oral and anal sides, and the double-stapling technique was employed to create the anastomosis (we administered 12.5 mg of ICG and confirmed that the anastomosis was contrasted in 27 s) (Fig. 7). The total operative time was 343 min, and the estimated amount of intraoperative blood loss was 72 ml. The patient developed postoperative paralytic ileus, which was resolved with conservative treatment. There were no postoperative impairments in blood flow in the lower extremities, and the patient was discharged on postoperative day 13. According to the UICC classification, 8th edition, the final pathological stage of disease was stage I (pT1N0M0). Recurrent cancer was not detected in the 6-month follow-up.

## Discussion

Leriche syndrome is a disease characterized by thrombotic occlusion in the aorta, frequently in the distal renal artery and classic symptoms of this syndrome include pain in the lower extremities emerging during activity, impalpability of femoral pulses, and impotency in male patients [1, 2]. Due to the chronic course of the disease, the collateral circulation is often well developed and ischemic symptoms are unlikely to appear [4]. In the present case, Leriche syndrome was found incidentally during a preoperative examination.

Two issues need to be considered when performing surgery for rectal cancer complicated by Leriche syndrome: anastomotic leakage due to decreased blood flow to the rectum caused by thrombotic occlusion of the internal iliac artery or IMA, and the risk of ischemia in the lower extremities due to intraoperative manipulations because blood flow to the lower extremities is supplied by collateral blood vessels. We employed the following two methods to treat anastomotic leakage. First, in addition to preserving the LCA, preoperative CT angiography was used to ensure that only the blood vessels feeding the tumor were treated in order to provide continuous blood flow to the rectum [5]. Although our patient had early-stage cancer, we consider that LCA preservation and IMA lymph node dissection should also be performed in advanced cancer cases instead of performing IMA lymph node dissection without preserving the LCA. The second method that we used was to evaluate the blood flow at the anastomotic site using ICG-based fluorescence imaging because such imaging has been reported to be useful for preventing anastomotic leakage [6]. Fluorescence imaging with ICG is also useful for selecting the optimal operative procedure in these cases because it objectively evaluates blood flow. If blood flow decreased in the present case, the procedure would have been converted to Hartmann’s operation. On the other hand, clinical trials that proposed the use of ICG fluorescence to reduce suture failure did not provide supportive evidence [7]. Therefore, mesenteric treatment also needs to be performed with considerations to secure blood flow and assess the color tone of the intestines. To avoid the risk of ischemia in the lower extremities, it is important to identify collateral blood vessels feeding the lower extremities and avoid intraoperative injuries. In patients with Leriche syndrome, the following two collateral pathways to the lower limbs are important: (i) the subclavian artery–internal thoracic artery–superior epigastric artery–inferior epigastric artery pathway and (ii) the subclavian artery–internal thoracic artery–lower intercostal or subcostal arteries–deep circumflex iliac artery pathway [3]. CT angiography may help surgeons to identify vascular variations preoperatively [8]. In the present case, the inferior epigastric artery functioned as a collateral pathway to the lower extremities. To prevent intraoperative injuries, we used magnified views when inserting the trocar, which helped us to avoid the inferior epigastric artery.

We encountered a case of rectal cancer complicated by Leriche syndrome, for which CT angiography was used to safely perform laparoscopic surgery. Since the incidence of arteriosclerosis is increasing, the number of patients with colorectal cancer complicated by Leriche syndrome is also expected to become higher. In these cases, it is important to use preoperative CT angiography to aid surgical planning.

## Conclusion

We report a case of safe surgery for rectal cancer complicated by Leriche syndrome using CT angiography and ICG-based fluorescence imaging.

## Data Availability

All data generated or analyzed during this study are included in this published article and its additional files.
